# Usability of a Novel Mobile Health iPad App by Vulnerable Populations

**DOI:** 10.2196/mhealth.7268

**Published:** 2017-04-11

**Authors:** David P Miller Jr, Kathryn E Weaver, L Doug Case, Donald Babcock, Donna Lawler, Nancy Denizard-Thompson, Michael P Pignone, John G Spangler

**Affiliations:** ^1^ Wake Forest School of Medicine Department of Internal Medicine Winston-Salem, NC United States; ^2^ Wake Forest School of Medicine Department of Social Sciences & Health Policy Winston-Salem, NC United States; ^3^ Wake Forest School of Medicine Department of Biostatistical Sciences Winston-Salem, NC United States; ^4^ Wake Forest Health Sciences Enterprise Information Management Winston-Salem, NC United States; ^5^ University of Texas Dell Medical School Department of Internal Medicine Austin, TX United States; ^6^ Wake Forest School of Medicine Department of Family & Community Medicine Winston-Salem, NC United States

**Keywords:** decision support techniques, technology assessment, primary care, health literacy, vulnerable populations

## Abstract

**Background:**

Recent advances in mobile technologies have created new opportunities to reach broadly into populations that are vulnerable to health disparities. However, mobile health (mHealth) strategies could paradoxically increase health disparities, if low socioeconomic status individuals lack the technical or literacy skills needed to navigate mHealth programs.

**Objective:**

The aim of this study was to determine whether patients from vulnerable populations could successfully navigate and complete an mHealth patient decision aid.

**Methods:**

We analyzed usability data from a randomized controlled trial of an iPad program designed to promote colorectal cancer (CRC) screening. The trial was conducted in six primary care practices and enrolled 450 patients, aged 50-74 years, who were due for CRC screening. The iPad program included a self-survey and randomly displayed either a screening decision aid or a video about diet and exercise. We measured participant ability to complete the program without assistance and participant-rated program usability.

**Results:**

Two-thirds of the participants (305/450) were members of a vulnerable population (limited health literacy, annual income < US $20,000, or black race). Over 92% (417/450) of the participants rated the program highly on all three usability items (90.8% for vulnerable participants vs 96.6% for nonvulnerable participants, *P*=.006). Only 6.9% (31/450) of the participants needed some assistance to complete the program. In multivariable logistic regression, being a member of a vulnerable population was not associated with needing assistance. Only older age, less use of text messaging (short message service, SMS), and lack of Internet use predicted needing assistance.

**Conclusions:**

Individuals who are vulnerable to health disparities can successfully use well-designed mHealth programs. Future research should investigate whether mHealth interventions can reduce health disparities.

## Introduction

Income, education, and race are powerful social determinants of health. Low socioeconomic status (SES) individuals and underrepresented minorities are at heightened risk for a variety of poor health outcomes, including shorter life expectancy and increased incidence of cancer and chronic diseases [1-4]. One pathway by which limited income and education, in particular, affect health negatively is by hampering the individuals’ ability to access, acquire, and understand health information needed to engage in preventive and self-care practices [5]. Some of this effect is mediated by lower levels of health literacy [5,6].

Recent advances in mobile technologies have created new opportunities to reach broadly into vulnerable populations, potentially decreasing informational barriers. Over the last 10 years, the growing ownership of cell phones, smartphones, and tablet devices has shrunk the digital divide. Over 90% of Americans own a cell phone with no significant differences seen by income, education, or race [7]. Additionally, two-thirds of Americans own a smartphone, including over half of adults with household incomes less than US $30,000 or only a high school education [7].

Many health care professionals are now using tablets or other mobile devices to assist patient care delivery [8,9], and there are a growing number of cell phone- and smartphone-based interventions published in the literature [10,11]. While the use of mobile health (or mHealth) strategies could decrease health disparities by better educating and empowering low SES individuals, they could also paradoxically increase health disparities if low SES individuals lack access to or the technical and literacy skills needed to use mHealth programs [12].

Colorectal cancer (CRC) is a source of health disparities. Individuals who are less educated, poorer, and members of minority populations are less likely to be screened for CRC and consequently, more likely to develop and die from CRC [4,13-15]. Therefore, we designed an mHealth patient decision aid about CRC screening specifically for use by individuals with limited resources and limited literacy skills.

If members of vulnerable populations experience greater difficulty using our mHealth program, our intervention could increase, rather than decrease, CRC-related health disparities. Indeed, prior studies found that members of vulnerable populations frequently encounter difficulties using Web-based or mHealth apps [16,17]. However, many of these previously studied apps assumed users have basic computer skills. Therefore, we sought to determine whether patients from populations vulnerable to health disparities could successfully navigate our program, which was designed under the assumption that users would have no prior experience with computers and would have difficulty reading. We analyzed baseline data from an ongoing randomized controlled trial (Trial ID NCT02088333) that is testing the effect of the intervention on completion of CRC screening. We compared usability metrics between patients vulnerable to health disparities (low income, limited health literacy, or black race) and other patients in the primary care setting.

## Methods

### Program Design

We designed a user-friendly mHealth iPad program for use by older individuals, many of whom we assumed would have little prior technology experience. Because over one-third of Americans have limited literacy skills, we also assumed many users would have reading difficulties [18]. We chose a touch screen interface, given its advantages over a mouse and keyboard. Touch-screen input mimics a user’s natural way of interacting with the world and requires less cognitive burden than manipulating external input devices [19]. Usability studies have demonstrated that older adults complete tasks more quickly and with less errors on touch-screen devices in comparison to using a computer mouse [20,21]. Moreover, novice and expert older users of touch-screen devices complete tasks with similar low error rates [22]. Some older adults also view touch-screen devices as less intimidating than computers [23].

**Figure 1:**
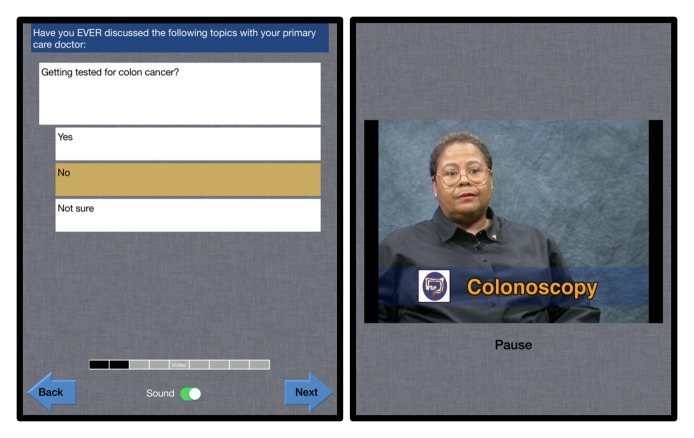
Sample screenshots from mobile Patient Technology for Health (mPATH) iPad program.

Our program, called mPATH (mobile Patient Technology for Health), begins with a self-administered survey to collect basic health information. Each screen displays a single question with large intuitive response buttons, as recommended by others (Figure 1) [19,24]. A narrator reads each question as well as the answer the user selects, both reducing literacy barriers and providing feedback that enhances usability [25]. The narrator also gives users basic instructions for navigating the program, such as instructing them how the “Back” and “Next” arrows function. Following the self-survey, the mPATH program displays a video about either CRC screening or healthy lifestyles, and then the program concludes with a short follow-up survey. All material in the program was written at the sixth grade level or below, which is a general recommendation for the development of patient education materials [26].

A team of experts in mobile app development, health literacy, and CRC screening developed the prototype, which was then refined based on pilot testing with a convenience sample of 40 primary care patients.

### Study Setting and Participant Recruitment

We enrolled English-speaking patients scheduled for a routine medical visit at one of six community-based primary care practices affiliated with a large academic medical center in North Carolina. All six practices shared a common electronic health record. We queried the electronic health record weekly to identify patients who were between the ages of 50 and 74 years and had no evidence of current CRC screening (colonoscopy within the last 10 years, flexible sigmoidoscopy within the last 5 years, or fecal testing for blood within the last 12 months).

We excluded patients who were already scheduled for a colonoscopy, were flagged as needing an interpreter, had a personal history of CRC, or had a potentially short life expectancy (receiving chemotherapy or radiation therapy for cancer within the last year, having advanced stage cancer, receiving hemodialysis, or being prescribed a medication for dementia). A research assistant called potentially eligible patients to inform them of the study and confirm their eligibility using a brief telephone survey. Additional study exclusion criteria assessed in the telephone eligibility survey included having a prior history of colon polyps, having a family history of CRC, and presence of rectal bleeding in the last month.

Eligible patients were asked to arrive at the clinic 45-60 minutes before their scheduled medical visit to enroll in the study and complete the mPATH iPad program. All participants provided a written informed consent, and the study was approved by the Wake Forest Health Sciences Institutional Review Board (IRB# 00023575).

### Study Procedures

The participants completed the mPATH program in a private location in the clinic immediately before their scheduled medical visit. They were given minimal instructions about how to use the program. The research assistant simply handed the participants the iPad, told them to touch the “start” button on the screen when they were ready to begin, and stated the narrator would walk them through the program. The research assistant then waited outside the room while the participants completed the program and instructed them to come to the door when they needed help using the program.

As described previously, the mPATH program begins with a 29-item self-administered survey. Then it randomly displays either a previously validated 8.7-minute CRC screening decision aid [27] or a 4.3-minute video about diet and exercise produced by the Center for Disease Control [28]. After the video, the program closes with another 35-item self-administered survey that includes 4 validated usability items [29]. The participants who viewed the CRC screening decision aid were shown an additional 1-4 items that allowed them to request a CRC screening test and sign up for follow-up text messages (short message service, SMS) or emails to support them through the screening process.

### Study Measures

The participants self-reported their race or ethnicity, cell phone ownership, use of the Internet, and use of SMS text messaging. We assessed health literacy using the validated item, “How confident are you filling out medical forms by yourself?” with responses varying on a 5-point Likert scale from “Extremely” to “Not at all” [30]. Consistent with published recommendations, individuals answering “Somewhat” (the midpoint) or less were defined as having limited health literacy [30]. We classified participants as members of a vulnerable population if they reported limited health literacy, annual household income < US $20,000, or black race. Races other than white or black comprised less than 4% of our study sample. Income was missing for 13 participants, and we classified those cases based only on race and health literacy.

Our primary outcome of interest was program usability, measured objectively and subjectively. Objectively, the research assistant counted the number of times a participant came to the door to ask for assistance to complete the program. The research assistant also recorded if a caregiver was present and helped the participant use the program. We measured the participants’ subjective rating of the program using three items from the System Usability Scale (ease of use, ease of learning to use the program, confidence using the program; scored on a 5-point Likert scale from strongly agree to strongly disagree) and an additional adjective rating of the overall user-friendliness (excellent, good, ok, poor, or awful) [29].

### Statistical Analysis

This study was designed to assess the impact of mPATH on 6-month CRC screening rates. The participants were randomly assigned with equal probability to receive within the mPATH program either the CRC screening decision aid or the diet and exercise video. A total sample size of 450 participants was required to detect a 12% absolute difference in screening rates between the two groups, with 80% power at the 5% two-sided level of significance assuming a 20% screening rate in the control group. The participants are still being followed for the primary objective; in this paper we review the baseline data associated with the usability of the mPATH program.

The participants were classified as being a member of a vulnerable population if they met the criteria described above. Time spent completing the mPATH program was calculated in minutes based on timestamps recorded by the iPad when the program began and when it ended. Chi-square tests (or Fisher exact tests) were used to assess the differences in user-friendliness and the usability scale items between those participants who were and were not classified as vulnerable. Needing assistance was dichotomized as none versus some, and chi-square tests were used to assess the association of this measure with demographic variables, health literacy, and technology use.

Logistic regression was used to determine whether being a member of a vulnerable population was associated with needing assistance after adjusting for other factors. Covariates included age, gender, owning a cell phone, Internet use, and frequency of texting. Separate logistic models included the components used to define vulnerability. To create more parsimonious models, we used a backward stepping algorithm removing any covariate that was not significant at a level < .20. All analyses were done using SAS, version 9.3 (SAS Institute, Inc); *P* values < .05 were considered significant.

## Results

### Participant Demographic Characteristics

Between June 2014 and May 2016, we enrolled 450 participants, all of whom completed the mPATH iPad program. Participant demographics are displayed in Table 1. Over two-thirds of the participants (305/450) were members of a vulnerable population; 36.9% had limited health literacy, 52.9% had annual incomes < US $20,000, and 37.6% were black. Many participants had not used the Internet in the last 30 days (36.0%), but 88.6% owned a cell phone.

**Table 1:** Sociodemographic and technology use characteristics of the participants enrolling in a colorectal cancer screening trial (N=450).

Characteristics		n (%)	
Female		242 (53.8)
Age in years, median (range)		57 (50-74)
Member of vulnerable population^a^		305 (67.8)
Limited health literacy		166 (36.9)
Annual household income < US $20,000 (n=437)		231 (52.9)
Black race		169 (37.6)
Own a cell phone (n=449)		398 (88.6)
**Frequency of texting^b^**		
	Daily or almost daily	219 (48.8)
	3-5 days per week	33 (7.3)
	1-2 days per week	41 (9.1)
	1-2 times per month	23 (5.1)
	Less than once per month	14 (3.1)
	Never	120 (26.7)
Used the Internet in the last 30 days (n=445)		285 (64.0)

^a^Vulnerable population=limited health literacy, annual income < US $20,000, or black race.

^b^How often a participant sends or receives a text message.

### Subjective Usability

The participants rated the overall user-friendliness of mPATH highly. Over 97% of both vulnerable and nonvulnerable participants rated the user-friendliness as “excellent” or “good” (Table 2). Similarly, over 90% of the participants in both groups moderately or strongly agreed with all three items of the System Usability Scale, although the percentage of participants who strongly agreed to each question was significantly lower in the vulnerable group (Table 2). Almost all participants from vulnerable and nonvulnerable groups stated they preferred the program over reading a brochure (97.7% and 95.2%, respectively, *P*=.15).

**Table 2:** Participant-rated usability of the mPATH mHealth program.

Usability rating			Vulnerable^a^ participants, n (%)	Nonvulnerable participants, n (%)	*P* value
Number of participants			305 (100)	145 (100)	
**Overall rating of user-friendliness**					.08
	Excellent		241 (79.0)	128 (88.3)	
	Good		56 (18.4)	16 (11.0)	
	OK		7 (2.3)	1 (0.7)	
	Poor		0 (0)	0 (0)	
	Awful		1 (0.3)	0 (0)	
**System Usability Scale items^b^**					
	The program was easy to use				.001
		Strongly agree	153 (50.2)	99 (68.3)	
		Agree	139 (45.6)	44 (30.3)	
		Neutral or less	13 (4.3)	2 (0.7)	
	Most people would learn to use the program very quickly				.008
		Strongly agree	137 (44.9)	82 (56.6)	
		Agree	152 (49.8)	62 (42.8)	
		Neutral or less	16 (5.2)	1 (0.7)	
	I felt very confident using the program				.009
		Strongly agree	148 (48.5)	92 (63.4)	
		Agree	146 (47.9)	51 (35.2)	
		Neutral or less	11 (3.6)	2 (1.4)	
	Strongly agree to all three questions		118 (38.7)	76 (52.4)	.006
	Agree to strongly agree to all three questions		277 (90.8)	140 (96.6)	.03
Prefer program over a brochure			298 (97.7)	138 (95.2)	.15

^a^Vulnerable population = limited health literacy, annual income < US $20,000, or black race.

^b^Each item is rated on a 5-point Likert-type scale ranging from strongly agree to strongly disagree.

### Objective Usability

The mean (standard deviation) time to complete the mPATH program was 22.8 (5.2) minutes for the CRC screening version (which included a few more survey items and a longer video), and 17.6 (4.6) minutes for the control version. Overall, adjusting for arm, the vulnerable group averaged 3.9 (0.46) minutes longer in completing the mPATH program (*P*<.001).

Only 6.9% (31/450) of the participants needed some assistance to complete the program (3.3% required only one episode of assistance, 2.0% required two or more episodes of assistance, and 1.6% had a caregiver help them use the program). The main reason that participants needed assistance was forgetting to touch the “Next” button to advance the program. A few participants became confused when they kept their finger too long on a phrase, which triggered the iPad to highlight the text. We prevented future occurrences of this user error by disabling the “copy and paste” native functionality of the iPad.

In unadjusted analyses, 9.5% (29/305) of vulnerable participants needed some assistance compared with 1.4% (2/145) of nonvulnerable participants (*P*<.01). Factors associated with needing assistance to complete the program in bivariate analyses included limited health literacy, low household income, older age, and less technology use (Table 3). Race was not associated with the need for assistance.

**Table 3:** Proportion of participants completing the mPATH mHealth program without any assistance.

Characteristics		n values (N=450)	n (%)	*P* value
**Health literacy level**				.004
	Limited	166	147 (88.6)	
	Normal	284	272 (95.8)	
**Annual household income**				.02
	< US $20,000	231	209 (90.5)	
	≥ US $20,000	206	198 (96.1)	
**Race**				.20
	Black	169	154 (91.1)	
	Nonblack	281	265 (94.3)	
**Vulnerable population^a^**				.002
	Yes	305	276 (90.5)	
	No	145	143 (98.6)	
**Gender**				.90
	Male	208	194 (93.3)	
	Female	242	225 (93.0)	
**Age in years**				<.001
	≤57	235	229 (97.4)	
	>57	215	190 (88.4)	
**Cell phone ownership**				.006
	Yes	398	376 (94.5)	
	No	51	43 (84.3)	
**Text messaging frequency**				<.001
	≥3 days per week	252	248 (98.4)	
	<3 days per week	198	171 (86.4)	
**Internet use in past 30 days**				<.001
	Yes	285	279 (97.9)	
	No	160	136 (85.0)	

^a^Vulnerable population = limited health literacy, annual income < US $20,000, or black race.

In a multivariable logistic regression model, being a member of a vulnerable population was no longer associated with needing assistance (*P*=.11). As vulnerable population was not significant, we looked at models that included the individual components that defined it (race, health literacy, and income). None of these components were statistically significant; only older age, less use of SMS text messaging, and lack of Internet use remained associated with needing assistance in both the full and reduced models (Table 4).

**Table 4:** Odds of needing assistance to complete the mPATH mHealth program by sociodemographic factors.

Factors	Full model OR^a^ (95% CI)	*P* value	Reduced model OR (95% CI)	*P* value
Texting <3 days per week	3.74 (1.12-12.5)	.033	4.05 (1.26-13.0)	.02
No Internet use in the past 30 days	3.63 (1.19-11.1)	.024	4.09 (1.50-11.1)	.006
Age >57 years	3.69 (1.39-9.80)	.009	3.63 (1.42-9.31)	.007
No cell phone ownership	1.08 (0.41-2.87)	.877	-^b^	
Limited health literacy	1.33 (0.57-3.10)	.515	-	
Black race	1.19 (0.50-2.87)	.693	-	
Annual income < US $20,000	1.01 (0.37-2.77)	.991	-	
Male gender	0.93 (0.41-2.14)	.868	-	

^a^OR: Odds ratio.

^b^Factor was removed from the reduced model by the backward stepping algorithm.

## Discussion

### Principal Findings

In this multisite study in which two-thirds of the participants were members of a low SES group or an underrepresented minority, over 90% of individuals were able to complete the mPATH iPad program without any assistance. Similarly, the participants rated ease-of-use very highly. In contrast, others have found that members of vulnerable populations frequently encounter difficulties in using Web-based or mHealth apps [16,17,31,32]. In contrast to our mPATH program, many of these apps require users to have advanced literacy, numeracy, and computer skills [17,33-36].

Our program’s ease-of-use is likely due to it being specifically designed for those with low health literacy and low computer literacy. We purposefully created a simple interface that displayed only one question per screen and used large response buttons, similar to what would be found at an automated teller machine or self-checkout kiosk. Likewise, we used simple language and included audio narration to assist those with literacy barriers. Other health apps with more complex navigational designs, denser text, and sophisticated terminology may explain the differences in usability observed.

Although low-income and low-literacy individuals were more likely to need help using the mPATH program in unadjusted analyses, this additional need for help disappeared after controlling for age, Internet use, and frequency of sending or receiving text messages. This indicates that older age and prior experiences with technology are drivers of usability, which is consistent with studies reporting that low-literacy and low-income individuals are less likely to use the Internet or own smartphones [7,37-39]. Relatedly, other studies have found that prior computer or Internet experience is associated with greater ease of use of health apps [33].

How age affects ease of use after accounting for differences in prior experiences with technology is less clear. We did not assess for the presence of health conditions that could affect usability, such as visual impairment, mild cognitive impairment, or conditions affecting dexterity. We also did not assess participants’ attitudes about technology, which could affect their confidence in using the program. In particular, computer anxiety may be a barrier for older adults [36,40,41]. Consequently, differences in these health conditions or attitudes may be responsible for the age-related differences in usability observed.

Although the participants with less technology experience were more likely to need help using the mPATH app, approximately six out of seven of these individuals were able to complete the program with no help at all. When the participants did require help, the most common reason for needing assistance was forgetting to press the “Next” button to advance the program. Simple changes to the design, such as highlighting the “Next” button to draw attention to it, could provide additional cues and increase usability further.

Although our results indicate that carefully designed mHealth programs can be used by vulnerable populations, care should be taken to ensure mHealth interventions do not increase health disparities [42-45]. The participants used our program on devices in the clinic setting. Cell phone ownership is consistent across socioeconomic strata, but an income- and education-related digital divide persists for smartphones and home broadband Internet access [7,46]. If the program was instead administered as a home app or on the Internet, low-income and low-literacy individuals would have less access. Similarly, the small differences in usability seen among older adults and those with less prior technology experience highlight the importance of ensuring apps are specifically designed for those who are computer naïve. Asking patients about use of the Internet or text messaging could be valuable screening questions for predicting who may have difficulty navigating mHealth programs.

### Limitations

Our study has limitations. Whereas we tested our program in several different clinic sites, we only included English-speaking patients. We also did not assess patients for specific health conditions that could impact usability (eg, vision impairment, hearing loss, paresis); usability in specific subpopulations could differ from what we observed. Finally, to decrease participant response burden, we included only a subset of items from the System Usability Scale.

### Future Work

Future studies should investigate which program features are most important for usability, and whether mHealth interventions can reduce health disparities. Results from our study examining the impact of mPATH on receipt of CRC screening will be forthcoming.

### Conclusions

In summary, we found that members of vulnerable populations could successfully use an mHealth program designed for individuals with limited literacy and technology skills. After controlling for other factors, literacy level and income did not predict usability. Race did not predict usability even in unadjusted analyses. These results indicate that properly designed mHealth interventions can reach broadly across populations.

## Abbreviations

CRC: colorectal cancer
mHealth: mobile health
mPATH: mobile Patient Technology for Health
SES: socioeconomic status
SMS: short message service
